# A Gly684Ala substitution in the androgen receptor is the cause for azoospermia in a Chinese family with mild androgen insensitivity syndrome and normal hormone levels

**DOI:** 10.3389/fgene.2022.988202

**Published:** 2022-09-09

**Authors:** Yuan Yuan, Wen-Qing Xu, Ying Chen, Tao Luo, Hou-Yang Chen

**Affiliations:** ^1^ Institute of Life Science and School of Life Science, Nanchang University, Nanchang, Jiangxi, China; ^2^ Key Laboratory of Reproductive Physiology and Pathology in Jiangxi Province, Nanchang, Jiangxi, China; ^3^ Reproductive Medical Center, Jiangxi Maternal and Child Health Hospital, Affiliated Maternal and Child Health Hospital of Nanchang University, Nanchang, Jiangxi, China

**Keywords:** androgen insensitivity syndrome, androgen receptor, azoospermia, hormone, infertility

## Abstract

Androgen receptor gene (AR) is essential for male growth and fertility. Its mutations are responsible for androgen insensitivity syndrome (AIS) that usually shows the phenotype of azoospermia resulting in male infertility. This study reported the first case of mild AIS with complete normal serum hormones in a Chinese family. The proband referred for infertility because of azoospermia. His uncle and two cousins are both infertile and have azoospermia. Whole-exome sequencing in the genetic analyses showed that the proband carries a novel hemizygous AR missense mutation, NM_000044.6: c.2051G>C (p.Gly684Ala), in exon four within the ligand-binding domain. His mother and maternal aunt are heterozygous carriers, while his father and brother are wildtype, indicating that the mutation in the proband was inherited from his mother. This pattern is consistent with the genetic model of the X-linked recessive inheritance of AR in AIS pathogenesis. HOPE predicts that p.Gly684Ala increases the hydrophobicity of AR but does not change the AR conformation. PolyPhen-2 predicts that p.Gly684Ala is harmful. This study provides the new knowledge to understand the *AR* gene mutations in MAIS.

## 1 Introduction

Androgen receptor (AR) binds androgen and plays a role in regulating male reproductive development (Bennett et al., 2010). The *AR* gene is located in Xq11-12 and encodes 920 amino acids, composed of the N-terminal transactivation domain (NTD), the DNA binding domain (DBD), and the C-terminal ligand binding domain (LBD) (Mongan et al., 2015). Mutations in the *AR* gene are the main reason of androgen insensitivity syndrome (AIS), which is divided into complete (CAIS), partial (PAIS), and mild (MAIS) according to the degree of feminization of patients (Bennett et al., 2010). Because a normal level of AR is necessary for responding to testosterone and gonadotrophin, which are essential for spermatogenesis, patients with AIS usually show the phenotype of male infertility (Hiort and Holterhus, 2003; Batista et al., 2018). In patients with MAIS who present with normal male external genitalia, infertility is often the only clinical manifestation of undervirilization (Hiort and Holterhus, 2003; Batista et al., 2018). However, the cause of male infertility in AIS remains unclear and the effectual remedy to obtain fertility in AIS patients has not been developed (Finlayson et al., 2017). But it was reported that the AIS patients would show the abnormal growth of reproductive organs and germ cells may be related to elevated oestrogens level caused by increased luteinizing hormone and testosterone (Hiort and Holterhus, 2003; Wang et al., 2009).

In this study, we reported the first case of MAIS with complete normal serum hormones in a Chinese family. A novel hemizygous AR missense mutation, NM_000044.6: c.2051G>C (p.Gly684Ala), in exon four within the ligand-binding domain were identified in the proband using whole-exome sequencing (WES). HOPE predicts that p.Gly684Ala increases the hydrophobicity of AR but does not change the AR conformation. PolyPhen-2 predicts that p.Gly684Ala is harmful. However, a previous study has shown that this mutation cannot affect the transactivation activity of AR when expressing this site-mutated *AR* in Hep3B cells (Koivisto et al., 1997). This study provides the new knowledge to understand the *AR* gene NM_000044.6: c.2051G>C mutations in MAIS.

## 2 Patients and methods

### 2.1 Case description

**FIGURE 1:**
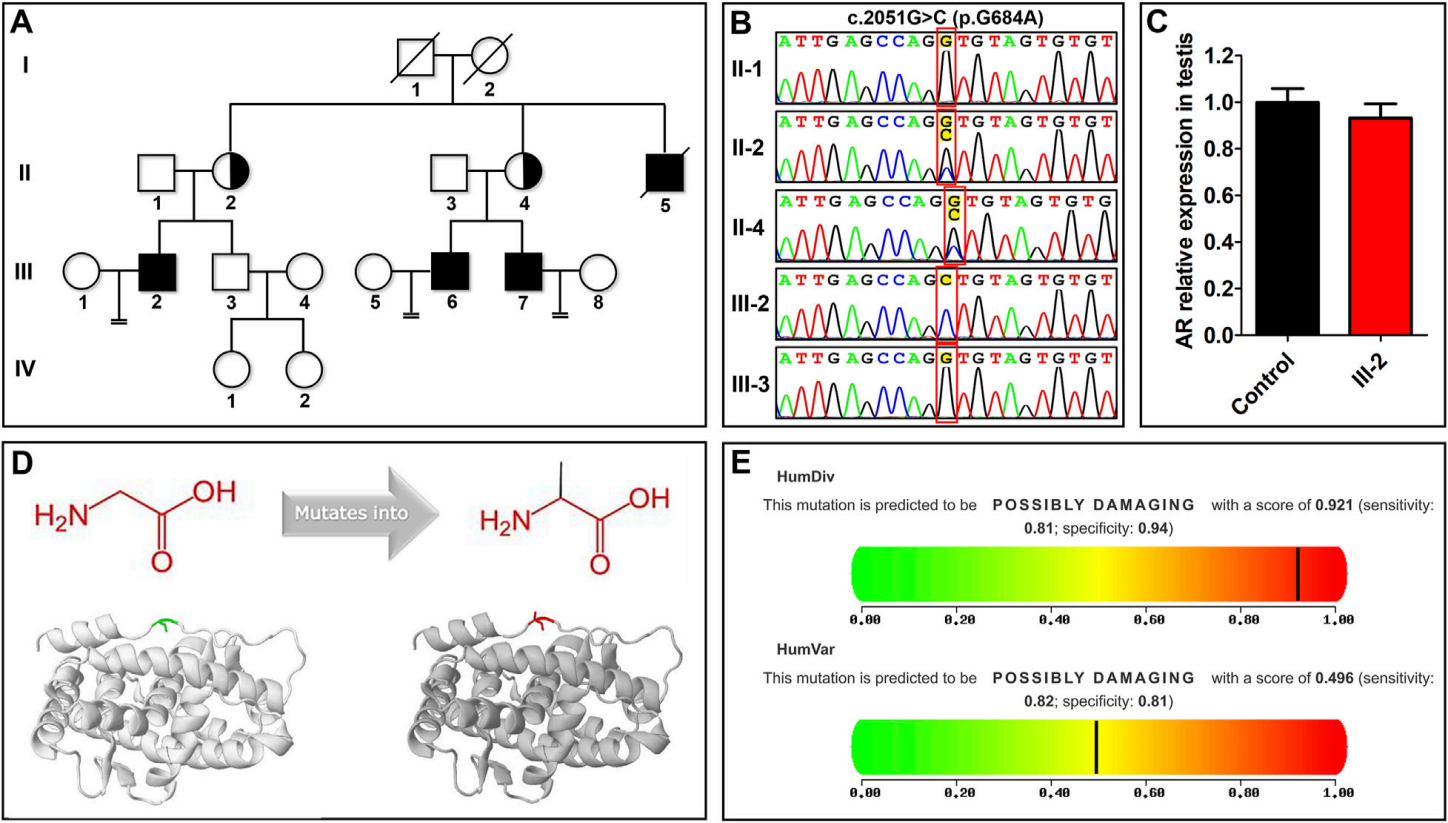
The nonsense mutation of the androgen receptor (*AR*) in a family with azoospermia. **(A)** A family with azoospermia was analyzed by whole-exome sequencing. A hemizygous *AR* missense mutation, NM_000044.6: c.2051G>C (p.G684A), was identified in the proband (III-2). **(B)** Sanger sequencing confirmed that the proband has a hemizygous mutation and his mother (II-2) and maternal aunt (II-4) are heterozygous carriers, while his father and brother are wildtype. **(C)** The relative mRNA expression levels of *AR* in testicular tissue from the proband is similar to a control subject. **(D)** The effect of the AR mutation on the protein structure was predicted by HOPE (https://www3.cmbi.umcn.nl/hope/). The amino acid is mutated from glycine to alanine; the gray part is the main part of the protein, the green part is the wild-type residue, and the red part is the side chain of the mutant residue mutation NM_000044.6: c.2051G>C (p.G684A). **(E)** The effect of the mutation on the organism was predicted by PolyPhen-2 (http://genetics.bwh.harvard.edu/pph2/). The result showed that the mutation is harmful.

A 32-year-old man was referred to the Reproductive Medical Center of Jiangxi Maternal and Child Health Hospital for evaluation of subfertility. The proband had been married for 10 years and had been unsuccessful in attempting to father a child. He had no significant medical or surgical history and was not on any long-term medications. Chromosomal analysis demonstrated a normal karyotype (46, XY). Y chromosome microdeletions were not detected in the proband. And the proband with normal hormone. The proband (III-2) is from a family with a history of infertility (Figure 1A). Semen analysis showed that no sperm are produced in the ejaculate (Table 1) and there are no germ cells in the testicular biopsies. His uncle (II-5) and two cousins (III-6 and III-7) are both infertile and have azoospermia. The family members participating in this study provided signed informed consent. This study was approved by the Institutional Ethics Committee on human subjects of Jiangxi Maternal and Child Health Hospital. Therefore, we employed whole-exome sequencing in the genetic analyses of the proband (III-2), his parents (II-1 and II-2), brother (III-3), and maternal aunt (II-4).

**TABLE 1:** Semen parameters and serum hormone levels in the proband carrying a hemizygous mutation in *AR*.

Parameters	III-2
Age (years)	32
Gene	*AR*
Genome location (GRCh37.p13)	chrX:186653613
DNA change (NM_000044)	Exon 4: c.G2051C (hemizygous)
Amino acid alteration (full length: 920 aa)	p.G684A (nonsense)
Allele frequency in gnomAD (%)	0
Semen volume (ml)	2.4 (Ref. > 1.5)
Total sperm (106/ml)	0 (Ref. > 39)
Sperm concentration (106/ml)	0 (Ref. > 15)
Follicle stimulating hormone (IU/L)	3.5 (Ref. 0.95–11.95)
Luteinizing hormone (IU/ml)	3.22 (Ref. 0.57–12.07)
Testosterone (ng/dl)	349.24 (Ref. 142.39–923.14)
Estradiol (pg/ml)	14.00 (Ref. 11–44)
Prolactin (ng/ml)	12.11 (Ref. 3.46–19.4)

### 2.2 Method

#### 2.2.1 Screening for candidate causative genetic variations

Genomic DNA was extracted from peripheral blood of each sample using the TIANamp Genomic DNA Kit (TIANGEN, Beijing). The Agilent SureSelect Human All ExonV6 Kit (Agilent Technologies, Santa Clara, CA, United States) and Illumina Novaseq 6,000 platform (Illumina Inc., San Diego, CA, United States) were used for exome capture and genomic DNA sequencing. The resulting fastq data were submitted to in-house quality control software to remove low-quality reads, and then were aligned to the reference human genome (hs37d5) using the Burrows–Wheeler Aligner (Li and Durbin, 2010). Single nucleotide variants (SNVs) and insertions/deletions (indels) were called with samtools to generate gVCF (Li et al., 2009). The raw calls of SNVs and indels were further filtered with the following inclusion thresholds: 1) read depth >4; 2) root-mean-square mapping quality of covering reads >30; and 3) a variant quality score >20. Annotation was performed using the ANNOVAR tool (Wang et al., 2010). Variants with a minor allele frequency >0.01 in 1,000 Genomes data (Genomes Project et al., 2015), esp6500siv2_all (https://evs.gs.washington.edu/EVS/), gnomAD data (Lek et al., 2016), and in house Novo-Zhonghua exome database from Novogene were filtered. Only SNVs occurring in exons or splice sites (within 10 bp splicing junctions) are further analyzed. Synonymous SNVs and small fragment non-frameshift (<10 bp) indels in the repeat region are discarded. Variations are screened according to scores of SIFT (Kumar et al., 2009), Polyphen (Adzhubei et al., 2010), MutationTaster (Schwarz et al., 2010), and CADD (Kircher et al., 2014), and the 787 potentially deleterious variations are reserved if the score of more than half of these four softwares support harmfulness of variations. The pathogenic variations were further predicted by the classification system of the American College of Medical Genetics and Genomics (Richards et al., 2015), there were three pathogenic variations, seven likely pathogenic variations and 1,179 uncertainty pathogenic variations. Given the characteristics of the pedigree, hemizygous, compound heterozygous, and *de novo* variants were considered to be candidate causal variations. The relationship between the proband and his parents was estimated using the pairwise identity-by-descent (IBD) calculation in PLINK (Purcell et al., 2007). The IBD sharing between the proband and parents in all trios is between 45% and 55%. Finally, AR and TEX11 be the candidate causative genetic variations.

#### 2.2.2 Real-time quantitative polymerase chain reaction

Total RNA extraction from testis tissue was extracted by Trizol (T9108, Takara), and cDNA was got by reverse transcription with RT kit (FSQ-101, TOYOBO). Taq II quantitative kit (DRR820A, Takara) was used to perform Real-time quantitative PCR with StepOnePlus™ Real-Time PCR System instrument (Thermo Fisher Scientific, Massachusetts, United States). Relative transcriptional levels of target genes was determined with the 2−^△△^Ct method.

#### 2.2.3 Protein structure predicted

Annotations about AR was obtained from UniProtKB entry P10275. HOPE SERVER(https://www3.cmbi.umcn.nl/hope/) was accessed to analyze the effect of variation (Venselaar et al., 2010). The structural information of human wild type Androgen Receptor was obtained from Protein Data Bank (PDB ID: 1E3G). And Missense3D (http://missense3d.bc.ic.ac.uk/∼missense3d/) was used to structure and assess missense variants (Ittisoponpisan et al., 2019).

#### 2.2.4 Statistical analyses

The means ± SEM of the data were expressed. T tests was used to assessed differences between the control and the sample.

## 3 Results

WES showed that the proband carries the hemizygous AR missense mutation NM_000044.6: c.2051G > C (p.G684A), which showed in Supplementary Material. His mother (II-2) and maternal aunt (II-4) are heterozygous carriers, while his father (II-1) and brother (III-3) are wildtype, indicating that the mutation of AR in the proband was inherited from his mother. This pattern is consistent with the genetic model of the X-linked recessive inheritance of AR in AIS pathogenesis. Combined with the score of more than half of these four softwares (SIFT, Polyphen, MutationTaster and CADD) support harmfulness of variations, we thought that NM_000044.6: c.2051G > C (p.G684A) was potentially deleterious variations. We also confirmed the variation in the individuals by Sanger sequencing (Figure 1B). Although this mutation is absent in the gnomAD browser (Table 1), it was previously found in the cancer cells of patients with prostate cancer (Koivisto et al., 1997). In this family, all the infertile males (the proband, his uncle II-5, and two cousins III-6 and III-7) did not have prostate cancer, which implies that c.2051G>C is not the causal mutation for prostate cancer. In addition, the proband has normal body hair, a normal male pattern facial hair, and normal male external genitalia (testicular volume of 18 ml, and penile measurements of 6.5 cm × 2.2 cm). In addition, infertility is the only clinical manifestation of undervirilization in his uncle (II-5) and two cousins (III-6 and III-7), without hypospadias or cryptorchidism. Therefore, this family represents a case of infertile MAIS.

However, it is unclear whether this mutation affects the function of AR in human testis. In this study, quantitative real-time polymerase chain reaction (PCR) showed that the testis of the proband transcribes an equivalent level of *AR* as that of a fertile control (Figure 1C). Therefore, HOPE and PolyPhen-2 were applied to predict the effect of the mutation on the AR protein, and Missense3D was used to predict the wildtype and mutation protein 3D structure (Figure 1D). HOPE and Missense3D predicts that p. G684A increases the hydrophobicity of AR but does not change the AR conformation (Figure 1D). PolyPhen-2 predicts that p. G684A is harmful (Figure 1E). These results suggest that p.G684A may affect the normal function of LBD, compromising the binding of AR to androgen.

## 4 Discussion

Around 600 AR mutations have been described in AIS, but there are few AR mutations related to MAIS, compared with CAIS and PAIS (Gottlieb et al., 2012). AR mutations in MAIS are mainly found in NTD and LBD of *AR* (Gottlieb et al., 2012). Intriguingly, the *AR* NM_000044.6: c.2051G > C mutation in this family occurred in exon 4, which encodes part of the LBD that binds to androgens and transmits downstream signals. On the other hand, the *AR* NM_000044.6: c.2051G > C mutation was consistent with that the reported MAIS were solely due to substitution mutations (Gottlieb et al., 2012). And the HOPE, Missense3D and PolyPhen-2 results (Figures 1D,E) suggest that p.G684A may affect the normal function of LBD, compromising the binding of AR to androgen. Androgen signaling in testis is essential for sperm production (Hughes et al., 2012). Therefore, the novel mutation in LBD of AR may be responsible for MAIS azoospermia.

In MAIS, hormone concentrations are usually normal, but elevated serum follicle stimulating hormone (FSH), luteinizing hormone (LH), and testosterone levels could be found in MAIS patients (Batista et al., 2018) (Karuppiah and Kudabadu, 2018). But Abou Alchamat et al. (2017) described, MAIS is often unnoticeable, but mainly detected in normal phenotypic males with infertility. Consistently, Peña et al. (2020) summarized that male infertility maybe the only pheotype of MIAS patients. The proband in this study has normal serum FSH, LH, estradiol (E2), prolactin (PRL), and testosterone (Table 1). It is reported that AR mutation caused the MAIS may interfere the spermatogenesis specific downstream actions but not affect recognize testosterone and not impaired the feedback on LH secretion, that maybe why the hormone is normal (Ochsenkühn and De Kretser, 2003). From this, the proband is the MAIS with complete normal serum hormones to our knowledge. According to this case, MAIS individuals with completely normal male sexual characteristics and without gynecomastia are likely to be diagnosed with infertility in the absence of genome sequencing. Therefore, genetic investigations serve as the precise diagnostic criterion for these MAIS patients (Zuccarello et al., 2008; Abou Alchamat et al., 2017).

Hormone replacement therapy has been used to treat AIS (Kosti et al., 2019), (Weidler et al., 2019). Two studies demonstrated that hormone therapy improves sperm count in infertile patients with AIS with AR mutations (p.V686A and p.N727K) and helps them become fathers (Yong et al., 1994; Tordjman et al., 2014). However, the hormone therapy did not improve the sperm count in an infertile male with MAIS with azoospermia and elevated serum FSH (Karuppiah and Kudabadu, 2018). In this study, we also performed hormone therapy for the proband, but he declined high-dose testosterone treatment. Whether and how the method of hormone replacement therapy is effective for patients with AIS with azoospermia requires verification. In addition, the proband received treatment with the traditional Chinese medicine Shengjing capsule, but it was not effective. Therefore, further study should be performed to develop the effectual remedy to obtain fertility in MAIS patients.

In conclusion, the present study reported that an infertile man with MAIS with azoospermia but normal serum hormone levels. The AR NM_000044.6: c.2051G>C mutation in this case is responsible for the phenotype of azoospermia independently of the transactivation activity of AR. This mutation provides a candidate target for studying the mechanism of AR in regulating spermatogenesis using the site-mutated mouse model.

## Data Availability

The datasets presented in this study can be found in online repositories. The names of the repository/repositories and accession number(s) can be found below: https://www.ncbi.nlm.nih.gov/bioproject/PRJNA867577.
